# Pulmonary thromboembolism exacerbated by thrombus migration through an inferior vena cava filter: a case report

**DOI:** 10.1093/ehjcr/ytag154

**Published:** 2026-03-05

**Authors:** Mari Kaneki, Masashi Koga, Yasuhiro Tanabe, Masaki Izumo, Yoshihiro J Akashi

**Affiliations:** Department of Cardiology, St.Marianna University School of Medicine, 2-16-1 Sugao, Miyamae-ku 216-8511, Kawasaki, Japan; Department of Cardiology, St.Marianna University School of Medicine, 2-16-1 Sugao, Miyamae-ku 216-8511, Kawasaki, Japan; Department of Cardiology, St.Marianna University School of Medicine, 2-16-1 Sugao, Miyamae-ku 216-8511, Kawasaki, Japan; Department of Cardiology, St.Marianna University School of Medicine, 2-16-1 Sugao, Miyamae-ku 216-8511, Kawasaki, Japan; Department of Cardiology, St.Marianna University School of Medicine, 2-16-1 Sugao, Miyamae-ku 216-8511, Kawasaki, Japan

**Keywords:** Pulmonary thromboembolism, Deep vein thrombosis, Surgical thrombectomy, Inferior vena cava filter, Case report

## Abstract

**Background:**

Pulmonary thromboembolism (PTE) is a life-threatening condition requiring prompt and effective treatment. Current treatment options include anticoagulation therapy, catheter-directed therapies, and surgical interventions. While inferior vena cava (IVC) filters are widely used for PTE prophylaxis in high-bleeding-risk patients, their failure mechanisms and optimal alternatives remain understudied, particularly in intermediate-high-risk PTE cases where anticoagulation fails.

**Case summary:**

We present a case of intermediate-to-high-risk PTE in a 61-year-old woman who experienced recurrent embolization despite receiving anticoagulation therapy. Although an IVC filter was deployed to prevent further embolization, a thrombus from the superficial femoral vein migrated through the tilted IVC filter, and surgical thrombectomy was performed.

**Discussion:**

This case emphasizes the need for careful IVC filter placement to prevent tilt-related complications and supports the adoption of advanced catheter-based thrombectomy devices to enhance the efficacy of PTE treatment and reduce the need for surgical intervention.

Learning points

- Malposition of an IVC filter leg into a lumbar vein can widen inter-strut spacing and compromise filtration, even with minimal tilt.
- Recurrent embolism despite adequate anticoagulation and filter placement warrants reassessment of filter position and possible filter failure.
- Restricted access to large-bore mechanical thrombectomy devices may necessitate surgical intervention when thrombolysis is contraindicated in intermediate-to-high-risk PTE.

## Introduction

Pulmonary thromboembolism (PTE) is a critical condition associated with significant morbidity and mortality if not promptly treated. Globally, its estimated annual incidence is 39–115 cases per 100 000 individuals, with mortality rates reaching 30% in untreated cases.^1^ Risk factors include prolonged immobilization, surgery, malignancy, and genetic predispositions. The incidence of PTE is increasing owing to an aging population and increasing rates of obesity and diabetes.^2^

PTE treatments are broadly categorized into three modalities: Medical therapy, catheter-based interventions, and surgery.^1^ Anticoagulation therapy remains the first-line treatment for most patients with acute PTE as it effectively reduces mortality and recurrence. However, if anticoagulation therapy is contraindicated or ineffective, catheter-directed therapy or surgery should be considered. Guidelines emphasize the importance of risk stratification and evaluation of therapeutic efficacy.^1^ Additional reperfusion strategies such as systemic thrombolysis, catheter-directed therapy, or surgical embolectomy should be considered for patients with haemodynamic instability. In patients with intermediate-to-high-risk PTE, additional reperfusion therapy is recommended only if anticoagulation therapy fails. Management may be further constrained in healthcare systems where access to advanced mechanical thrombectomy devices remains limited.

We present a case of intermediate-to-high-risk PTE that illustrates the challenges of managing recurrent embolization with currently available treatment options and highlights the potential benefits of advanced catheter-based therapies.

## Summary figure

**Figure:**
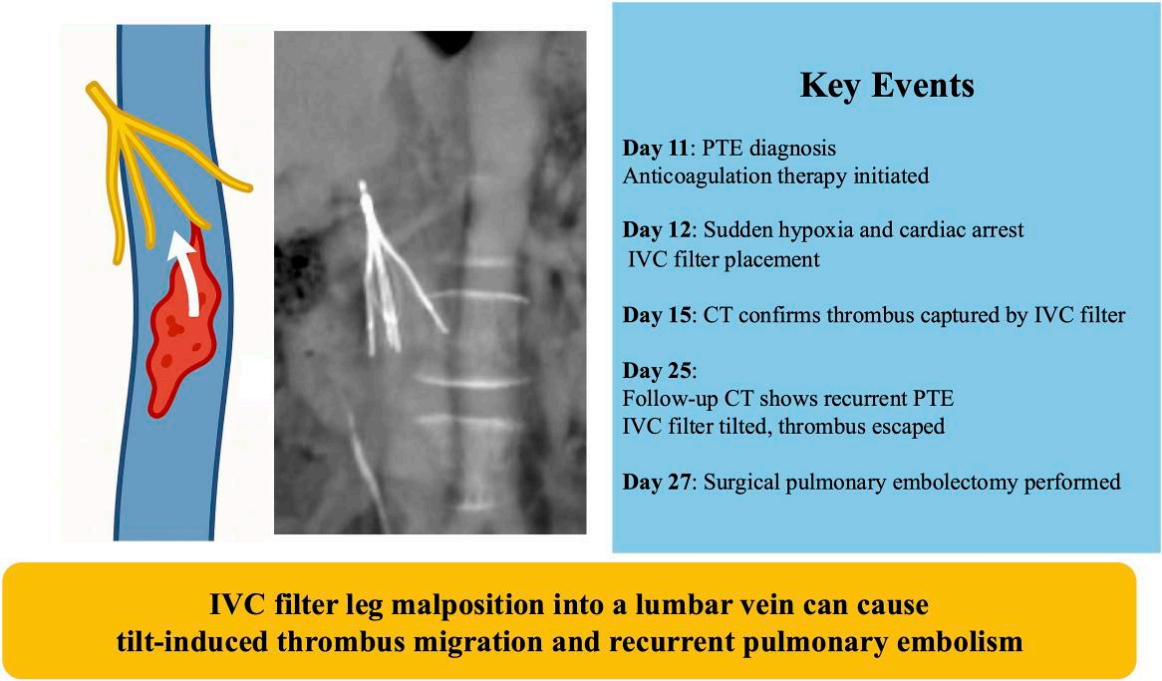


## Case presentation

**Figure 1:**
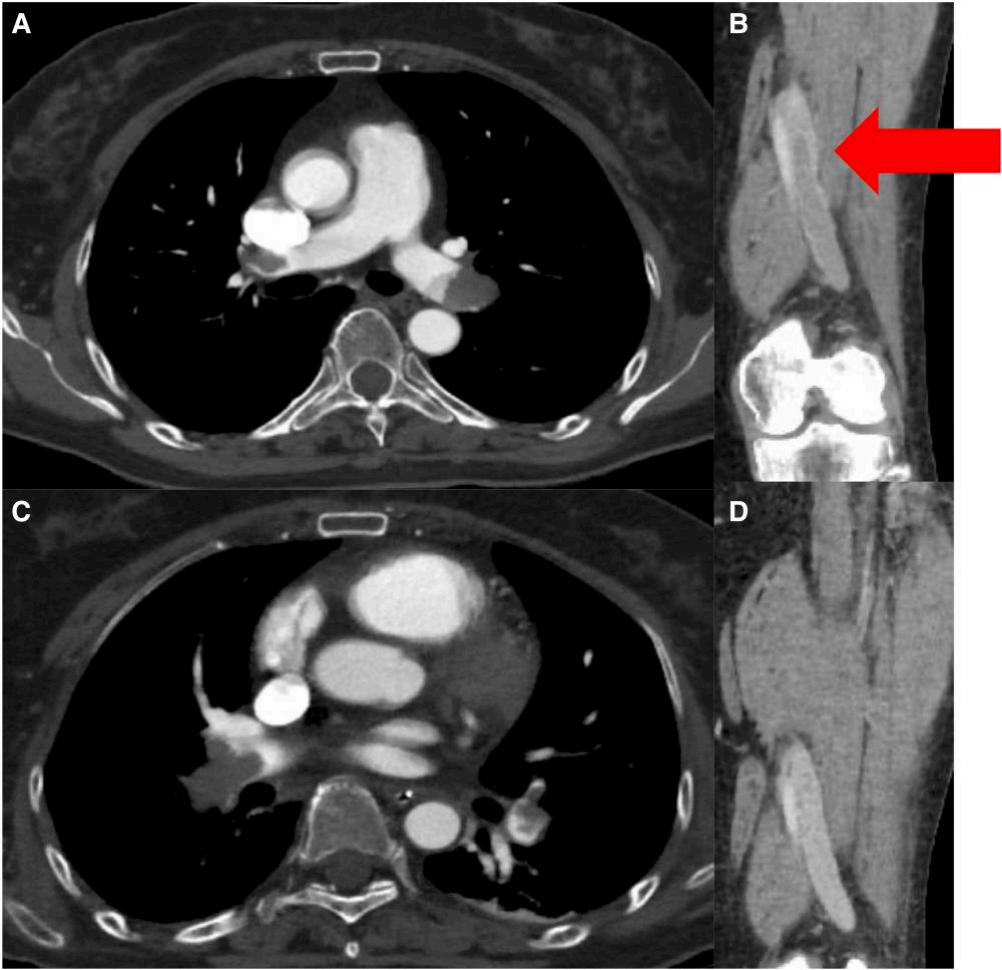
Time course of the pulmonary embolism and deep vein thrombosis (*A* and *B*) contrast-enhanced computed tomography reveals bilateral pulmonary embolism and deep vein thrombosis in the left superficial femoral vein (arrow). (*C* and *D*) Follow-up enhanced computed tomography demonstrates a large thrombus in the right pulmonary artery and resolution of the deep vein thrombosis in the left superficial femoral vein.

A 61-year-old woman presented with headaches, nausea, and difficulty walking. She was diagnosed with a right falcine meningioma. While waiting for neurosurgical intervention, dyspnoea and elevated D-dimer levels were observed. On hospital day 11, contrast-enhanced computed tomography (CT) revealed bilateral pulmonary embolisms (*Figure 1A*) and deep vein thrombosis (DVT) of the left superficial femoral vein (*Figure 1B*), prompting referral to our department. No prophylactic anticoagulation had been administered prior to diagnosis.

On admission, the patient was tachycardic (heart rate 127 bpm) and hypoxemic (oxygen saturation 94% on 1 L/min supplemental oxygen), while remaining haemodynamically stable. Laboratory investigations revealed elevated D-dimer (21.0 µg/mL), NT-proBNP (642 pg/mL), and troponin T (0.171 ng/mL) levels. Screening for thrombophilia was negative. Electrocardiography showed sinus tachycardia with T-wave inversions in leads III, aVF, and V1–V2. Transthoracic echocardiography revealed a D-shaped left ventricle and McConnell’s sign, right ventricular dysfunction (decreased right ventricular fractional area change: 24%), and pulmonary hypertension [elevated right ventricular systolic pressure (RVSP): 42 mmHg].

**Figure 2:**
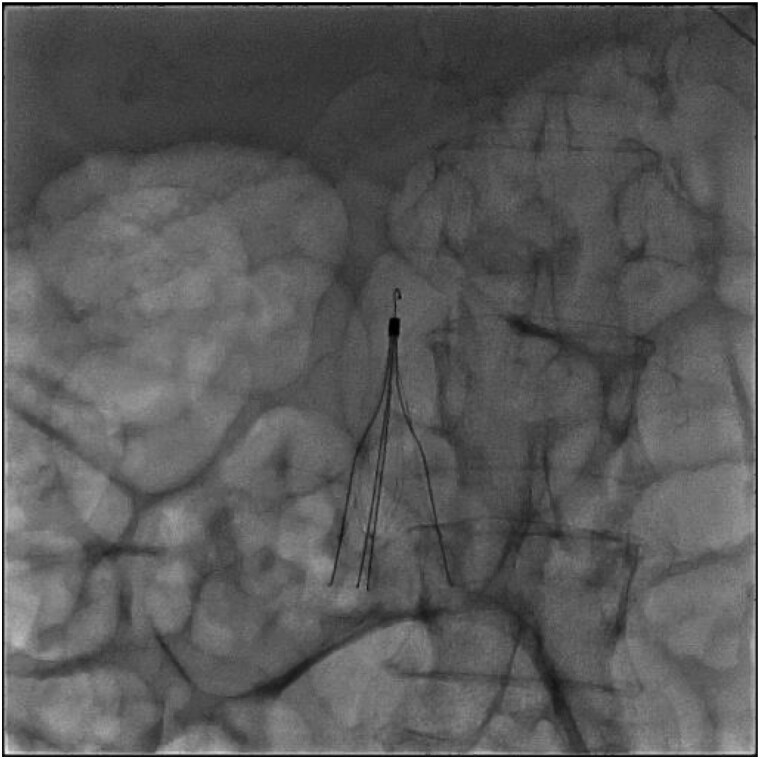
X-ray fluoroscopy showing placement of an inferior vena cava filter for the prevention of recurrent embolism.

**Figure 3:**
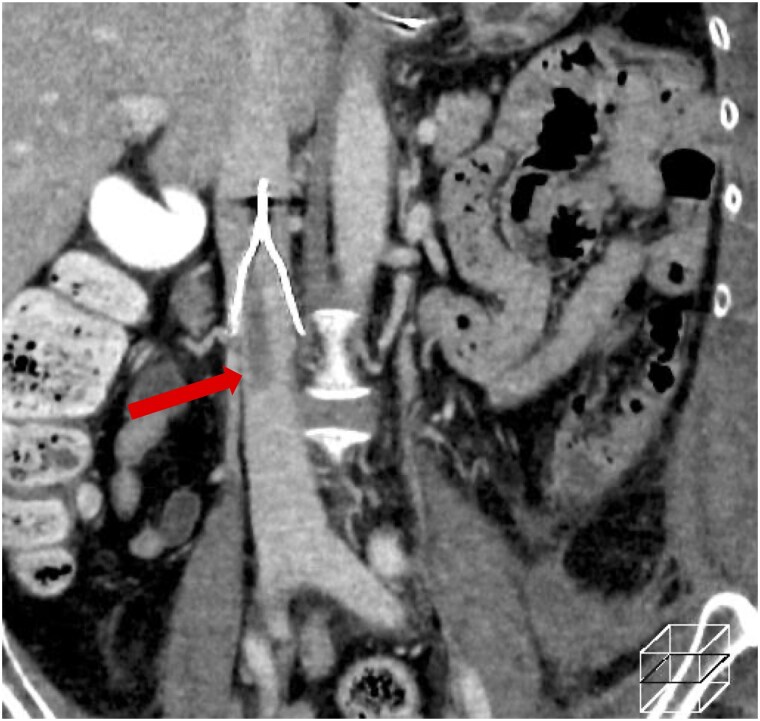
Contrast-enhanced computed tomography confirms capture of the thrombus within the inferior vena cava filter (arrow).

**Figure 4:**
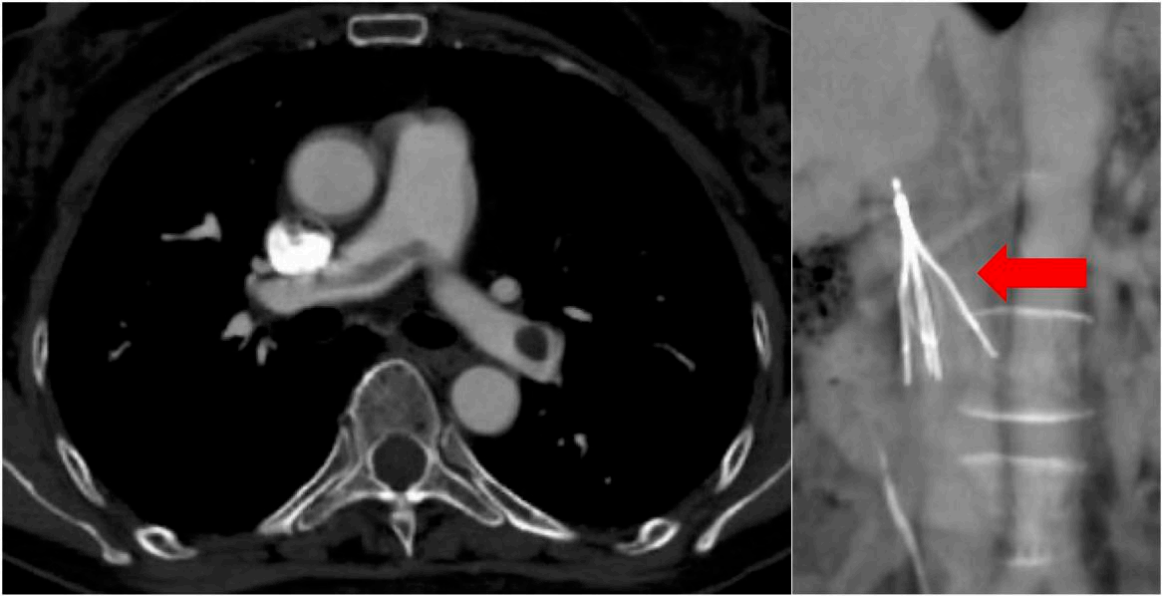
Contrast-enhanced computed tomography reveals a large thrombus in the pulmonary artery, and the previously observed thrombus in the inferior vena cava filter has disappeared. Additionally, the inferior vena cava filter is tilted.

**Figure 5:**
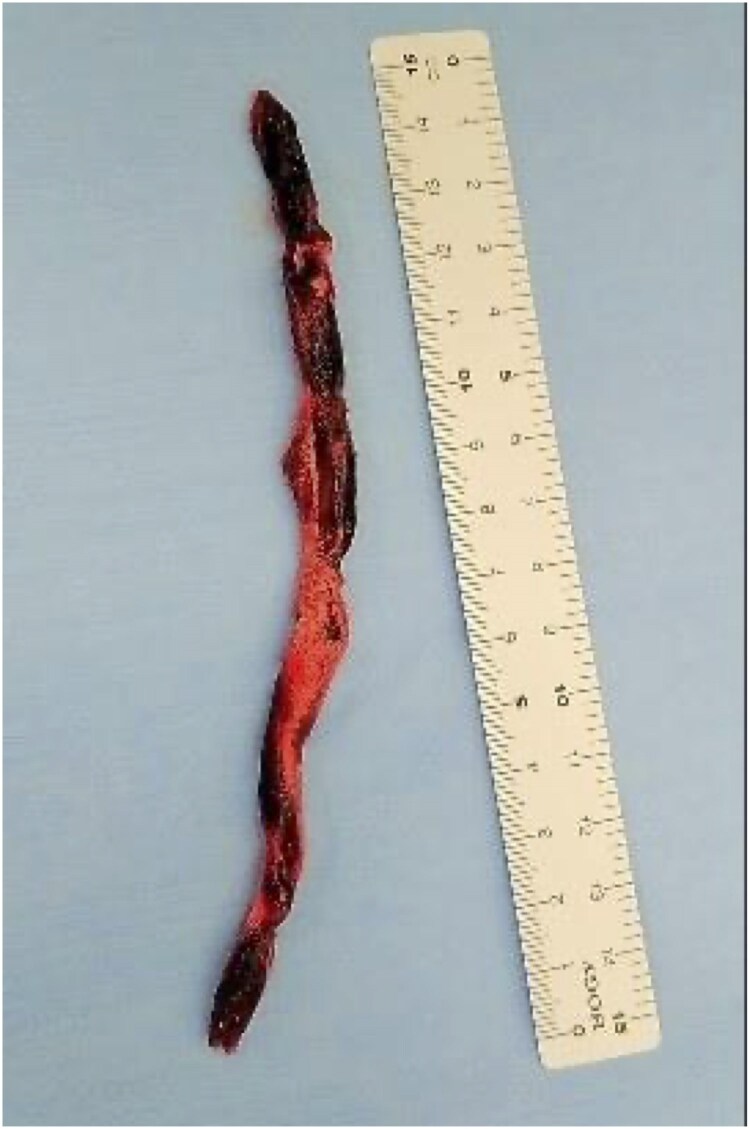
Photograph of the surgically excised thrombus, showing an organizing component with adherent fresh thrombus.

She was diagnosed with intermediate-to-high-risk PTE and proximal DVT, and anticoagulation therapy with unfractionated heparin was administered, targeting an activated partial thromboplastin time of 60–80 s (range, 25–40 s). On the following day (hospital day 12), she acutely developed hypoxaemia and hypotension, progressed to cardiopulmonary arrest, and was promptly and successfully resuscitated. On enhanced CT, extension of the thrombus in the right pulmonary artery (*Figure 1C*) and partial disappearance of a thrombus previously seen from the left SFV to the popliteal vein were observed (*Figure 1D*), suggesting the tip of the thrombus in the left SFV had dispersed into the right main pulmonary artery. Systemic thrombolysis with a tissue plasminogen activator was contraindicated owing to the presence of an intracranial tumour. The patient was managed in the intensive care unit with anticoagulation therapy. Ultrasonography revealed a floating thrombus in the femoral vein. Owing to significant right ventricular overload (RVSP 47 mmHg), further embolization of the floating thrombus was considered potentially fatal. Therefore, an inferior vena cava (IVC) filter was inserted (*Figure 2*). On hospital day 15, contrast CT confirmed that the thrombus was captured by the IVC filter (*Figure 3*). Rivaroxaban was initiated at 15 mg twice daily on hospital day 20. However, on hospital day 25, follow-up contrast CT revealed a large thrombus in the pulmonary artery. The thrombus in the IVC filter had disappeared, suggesting that the thrombus captured by the IVC filter had migrated (*Figure 4*). Additionally, the IVC filter was notably tilted because of leg misplacement in the L3 lumbar vein. At that time, right ventricular dilatation had improved, RVSP had decreased to 26 mmHg, and McConnell’s sign was no longer evident. After discussion with the heart team, surgical pulmonary embolectomy was performed on hospital day 27. The explanted thrombus consisted of a white-toned organizing clot with a dark-red fresh thrombus (*Figure 5*). Follow-up contrast-enhanced CT after surgical pulmonary embolectomy showed complete resolution of the pulmonary artery thrombi, with residual thrombosis confined to the distal veins below the left knee; therefore, the IVC filter was subsequently removed. High-dose rivaroxaban was continued for 3 weeks and then reduced to the standard maintenance dose. On hospital day 46, the patient was transferred to the neurosurgery department, where embolization of the tumour-feeding arteries was performed for the right falcine meningioma. Contrast-enhanced CT at the 9-month follow-up showed no residual thrombus. Given the life-threatening nature of the event, lifelong anticoagulation was continued despite removal of the initial provoking factor.

## Discussion

This case highlights both the limitations of current treatments in certain regions and the risks associated with IVC filters. The IVC filter captured additional emboli during the acute phase, preventing haemodynamic collapse. However, subsequent filter tilting allowed thrombus migration, leading to a saddle pulmonary embolism in the main pulmonary artery trunk that necessitated surgical intervention. Although reduced efficiency of tilted IVC filters in preventing PTE has been previously reported,^3^ in this case the underlying mechanism was directly visualized using contrast-enhanced CT.

In this case of intermediate-to-high-risk PTE with right ventricular overload and a large thrombus burden, systemic thrombolysis was contraindicated due to an intracranial tumour, and anticoagulation was selected as initial therapy. Unfractionated heparin was used due to local formulary restrictions. Following cardiopulmonary arrest, an IVC filter was placed for a persistent floating femoral thrombus and successfully prevented acute re-embolization; however, recurrent pulmonary embolism occurred in the subacute phase due to thrombus migration through a tilted filter. Despite improvement in right ventricular function, surgical embolectomy was indicated because a 15 cm saddle thrombus at the pulmonary bifurcation posed a risk of fragmentation, and recurrence under therapeutic anticoagulation suggested treatment resistance. Surgery was elected to prevent haemodynamic deterioration and progression to chronic thromboembolic pulmonary hypertension. Systemic thrombolysis was contraindicated owing to the presence of an intracranial tumour and recent cardiopulmonary resuscitation. In this clinical setting, catheter-based embolectomy devices were unavailable, and thrombus fragmentation techniques (e.g. balloons or pigtail catheters), which are occasionally performed when other therapies are contraindicated, may cause embolic material to scatter into the peripheral pulmonary arteries, leading to an increase in pulmonary vascular resistance and subsequent haemodynamic deterioration.^4,5^ Internationally, newer-generation catheter-directed devices with larger diameters, such as the INARI FlowTriever®(Inari Medical, Irvine, California) and Penumbra Indigo®(Penumbra Inc., Alameda, California), have demonstrated efficacy in thrombus removal and right ventricular unloading without thrombolytic agents.^6,7^ However, these advanced therapeutic options remain inaccessible to certain healthcare systems.

Numerous reports have documented complications associated with both permanent and retrieval IVC filters, such as filter occlusion, migration, IVC stenosis, and DVT recurrence.^8–11^ In this case, although the IVC filter was initially well positioned, venous peristalsis during the clinical course likely caused one filter leg to migrate into and become fixed within the L3 lumbar vein. This malposition generated asymmetric forces, resulting in filter tilt and widening of the inter-strut spaces (Summary Figure). As a possible contributing factor, the thrombus initially captured within the filter may have decreased in size under anticoagulation, facilitating passage through the filter. However, because contrast-enhanced CT demonstrated that the thrombus straddling the main pulmonary artery was comparable in size to the thrombus previously captured by the filter, we believe that filter tilt and enlargement of the inter-strut gaps due to leg malposition into the L3 lumbar vein were the primary mechanisms permitting thrombus migration. Although a tilt angle ≥15° has been associated with reduced prophylactic efficacy and increased retrieval difficulty,^12^ this case suggests that even a modest tilt of ∼6°, when accompanied by single-leg malposition, may substantially compromise filter function. In our clinical setting, the Günther Tulip® (Cook, Bloomington, Indiana) and Denali™ (Bard Peripheral Vascular, Inc, Tempe, Arizona) filters are currently available, and their designs differ substantially. The Denali™ filter is equipped with six short arms, which is designed to reduce the likelihood of filter tilt, although fractures following long-term implantation have been reported.^13,14^ In this patient, uncertainty regarding long-term anticoagulation due to an intracranial tumour and the potential need for prolonged filter placement led to selection of the Günther Tulip® filter. Nevertheless, regardless of device choice, meticulous attention during implantation is essential to avoid lumbar vein leg malposition and minimize filter tilt. When tilt is observed, the risk of thrombus passage through the filter should be carefully considered.

## Data Availability

Non-identifiable data underlying this article will be made available upon reasonable request to the corresponding author.

## Lead author biography

Mari Kaneki, MD, is a clinical fellow in the Department of Cardiology at St. Marianna University School of Medicine, Japan. Her areas of interest include coronary and structural heart interventions, as well as thromboembolic disorders.

## Notes

**Consent:** The authors confirm that written consent for the submission and publication of this case report, including images and associated text, has been obtained from the patient in accordance with COPE guidance.
